# Identification of a gene signature for discriminating metastatic from primary melanoma using a molecular interaction network approach

**DOI:** 10.1038/s41598-017-17330-0

**Published:** 2017-12-11

**Authors:** Rahul Metri, Abhilash Mohan, Jérémie Nsengimana, Joanna Pozniak, Carmen Molina-Paris, Julia Newton-Bishop, David Bishop, Nagasuma Chandra

**Affiliations:** 1 IISc Mathematics Initiative (IMI), Indian Institute of Science, Bangalore, Karnataka India; 2 Department of Biochemistry, Indian Institute of Science, Bangalore, Karnataka India; 3 Section of Epidemiology and Biostatistics, Leeds Institute of Cancer and Pathology, University of Leeds, Leeds, UK; 4 Department of Applied Mathematics, School of Mathematics, University of Leeds, Leeds, UK

## Abstract

Understanding the biological factors that are characteristic of metastasis in melanoma remains a key approach to improving treatment. In this study, we seek to identify a gene signature of metastatic melanoma. We configured a new network-based computational pipeline, combined with a machine learning method, to mine publicly available transcriptomic data from melanoma patient samples. Our method is unbiased and scans a genome-wide protein-protein interaction network using a novel formulation for network scoring. Using this, we identify the most influential, differentially expressed nodes in metastatic as compared to primary melanoma. We evaluated the shortlisted genes by a machine learning method to rank them by their discriminatory capacities. From this, we identified a panel of 6 genes, *ALDH1A1*, *HSP90AB1*, *KIT*, *KRT16*, *SPRR3* and *TMEM45B* whose expression values discriminated metastatic from primary melanoma (87% classification accuracy). In an independent transcriptomic data set derived from 703 primary melanomas, we showed that all six genes were significant in predicting melanoma specific survival (MSS) in a univariate analysis, which was also consistent with AJCC staging. Further, 3 of these genes, *HSP90AB1*, *SPRR3* and *KRT16* remained significant predictors of MSS in a joint analysis (HR = 2.3, P = 0.03) although, *HSP90AB1* (HR = 1.9, P = 2 × 10^−4^) alone remained predictive after adjusting for clinical predictors.

## Introduction

Malignant melanoma, a cancer arising from the melanocytes is reported to have one of the largest rates of increase in incidence worldwide^1,2^. According to the World Health Organization, current statistics indicate that 132,000 cases occur globally each year^3^. The majority of primary tumours are cured by local excision^4^ but the trend towards increased numbers of tumours in older males (age and male sex^5^ being risk factors for melanoma death) suggests that metastatic AJCC stage IV melanoma will continue to increase in incidence. Although the advent of targeted therapies, such as *BRAF* inhibitors and checkpoint therapies have for the first time produced a survival advantage, long term survival is still only seen in around 20% of patients^6^. Improvement is therefore necessary both in its detection and in its treatment. Computational methods are necessary for an unbiased comprehensive analysis so as to identify the characterizing genes of the metastatic phenotype.

Genome sequencing and analysis by The Cancer Genome Atlas (TCGA) has led to the identification of driver mutations in around 70% of tumours and a classification of patients into *BRAF*, *NRAS*, *NF1* and *tripleWT* subtypes^7^. In addition, other studies have identified a series of mutations or copy number changes^8^. These have provided insights into the underlying molecular mechanisms but have little prognostic or diagnostic significance. Many of the currently available markers (*TYR*, *HMGA2*, *TRIB2*, *MITF* and *PMEL*) depend on the differential expression of these markers in the diseased state^9–13^. Although there was previously little agreement between transcriptomic studies in melanoma, we recently replicated signatures described by Jonsson’s group^14,15^ giving rise to the view that increasing study size, better platforms and bioinformatics may now lead to the identification of better biomarkers.

The most common analytical tools used for biomarker identification are clustering methods^16^, classification using a support vector machine^17^, decision trees and random forest classifiers^18,19^, artificial neural networks^20^ and simple differential expression based analysis^13,21^. These methods are typically data-driven and do not consider any biological information of the component genes as an input, but have an advantage of identifying distinguishing features even when no information is available about that feature. On the other hand, biological networks, constructed on the basis of the known functions and interactions of individual molecules, offer alternate approaches that are superior to blind learning approaches. Networks have the added advantage of combining condition specific transcriptome data and allow understanding of the functional role of the individual genes capable of discriminating disease from healthy or between different disease stages^22–24^. Machine learning methods on the other hand are capable of providing a quantitative picture of the classification efficiencies of the individual genes^25,26^. They fail when the number of features is higher than the number of samples. To get the best of both approaches, we have combined the two and used network analysis which facilitates the usage of machine learning methods by reducing the number of features to be tested for classification efficiency and derive the final signature. This type of a combination approach has been suggested earlier to yield the best classification as compared to individual methods alone^27^. Initially, a genome-scale molecular interaction network was rendered condition-specific by integrating transcriptome data. Next, we mined the networks to identify a shortlist of key components that would define the state of tumour, progression stages and key points of perturbation. We then used a machine learning method to derive different signatures with an optimal length to discriminate primary and metastatic melanoma, respectively. We then went on to validate the signature genes based on Melanoma specific survival (MSS) analysis from an independent cohort.

## Results and Discussion

### Biomarker identification strategy

**Figure 1:**
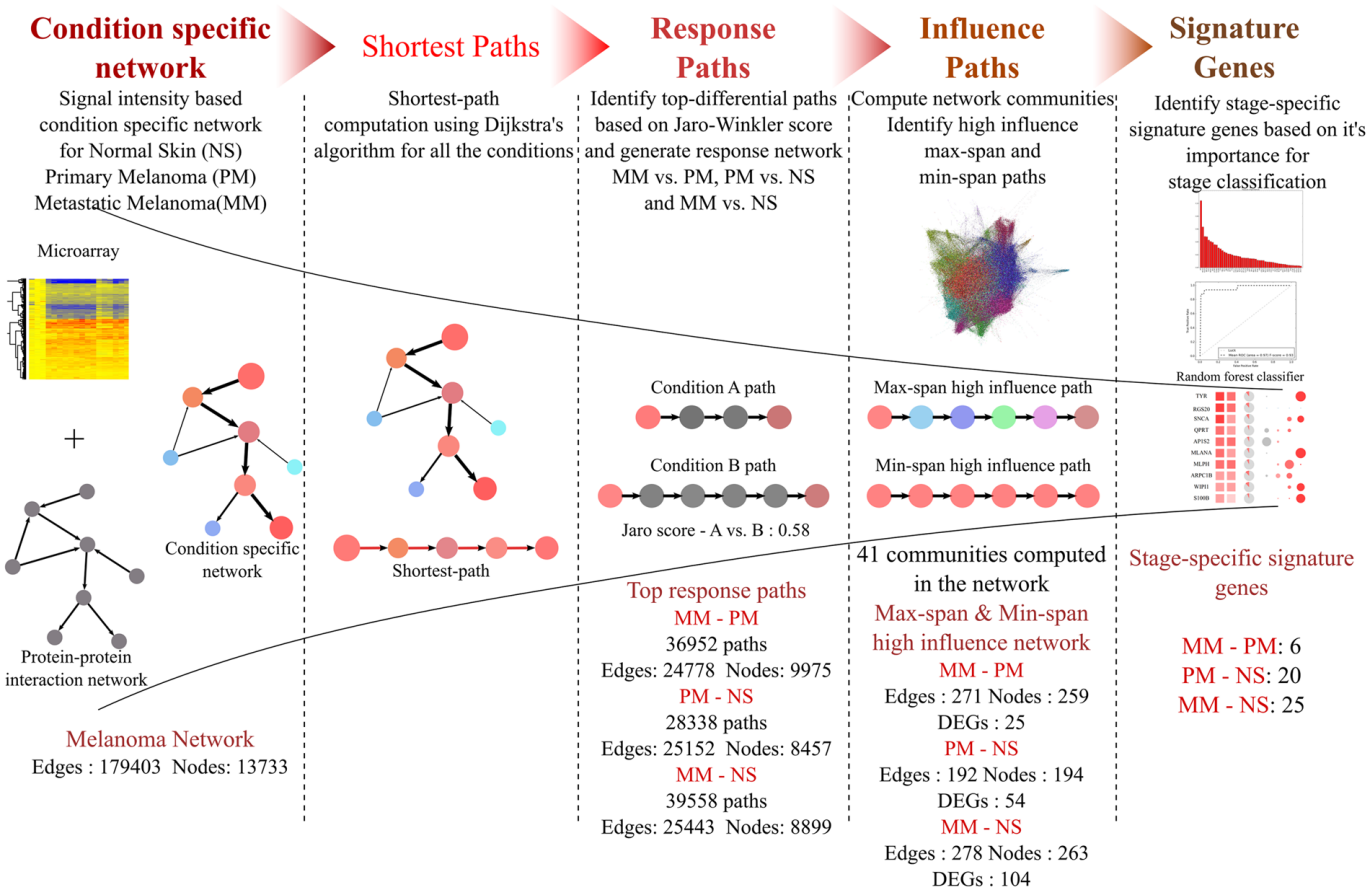
A schematic representation of the biomarker identification pipeline. The pipeline involves 5 major steps. Condition specific network: Construction of weighted network using protein-protein interaction network and gene expression data. Shortest Path analysis: Identification of all-vs.-all nodes shortest paths using Dijkstra’s algorithm. Response Paths: Paths with highest differential activity in diseased condition identified using string matching metric. Influence paths: Prioritizing paths based on influence on the network. Signature genes: Feature ranking of genes to obtain minimal set classifying conditions. The conditions considered for study: Normal Skin (NS), Primary Melanoma (PM) and Metastasis Melanoma (MM). Analysis results in numbers is shown in bottom section of the image

We configured a pipeline to identify RNA based biomarker candidates distinguishing metastatic melanoma from primary melanoma in an unbiased fashion using well established methods at each step. As illustrated in Fig. 1, the pipeline (a) begins with the reconstruction of knowledge-based protein-protein interaction networks. (b) These are then rendered condition-specific by weighing the network based on fitted signal intensity values resulting in three different networks for NS, PM and MM respectively, using methods previously established by us^28,29^. (c) The PM and MM networks were either compared with each other or compared with NS to generate disease response networks. For this, the highest activity paths were compared for a given pair of conditions and the top-ranked perturbed paths shortlisted for inclusion in further steps. The selected set of such paths in each comparison were found to be well connected with each other and hence form the corresponding response networks. (d) This is followed by identification of high-influence paths based on the paths impact on the network by constructing network communities using standard graph theory approaches. (e) Highest ranked influential paths and genes in them were then used as an input into a machine learning classifier that yields a final signature that discriminated metastatic from primary melanoma and predicts the risk of disease progression in primary melanoma.

Among the publicly available transcriptome repositories for cutaneous melanoma, we initially selected a transcriptional profiling dataset that contained data for tissue samples of primary melanoma, metastatic melanoma along with adjacent normal skin^10^. We used this to identify biomarker signatures to distinguish between (a) metastatic and primary melanoma (b) metastatic melanoma and normal skin (c) primary melanoma and normal skin. The shortlist of possible biomarker candidates was obtained and evaluated for the performance of the signatures on an independent dataset for the first phase of validation and used it for pruning the candidate set, thereby deriving an optimal signature. For the next phase of fully independent validation, we evaluated the performance of the optimized biomarker panel using a large dataset from the Leeds Melanoma^30^ cohort for which survival information was available.

### Response networks capture disease stage-specific variations in an unbiased fashion

We utilized a comprehensive master network of interactions between human proteins previously constructed in the laboratory^28^ (Methods). The master network comprises 13733 proteins (nodes) connected by 179403 interactions (edges) and includes both structural as well as functional interactions, belonging to several signaling, metabolic and regulatory processes, thus providing a global coverage of the human protein interactome. We rendered the master network condition-specific by weighting the individual nodes proportional to their respective fitted gene expression intensities from the transcriptomes of 46 and 12 samples of primary melanoma (PM) and metastatic melanoma (MM), respectively^10^. The dataset also contained 16 normal skin (NS) samples. A transcriptome comparison of MM vs. PM indicated 925 differentially expressed genes (DEGs, adjusted p-value ≤ 0.05, fold change ≥ 2). The same comparison for PM vs. NS is in the order of 2739 DEGs while that in MM vs. NS are 4262. A majority of DEGs (72% of MM vs. PM) were present in the initial network, indicating that the network has high coverage of the variations in melanoma and a similar trend was observed in other comparisons as well. From the three condition-specific networks reflecting conditions of NS, PM and MM, we obtained shortest paths by computing paths for all-vs.-all node pairs in each weighted network.

**Figure 2:**
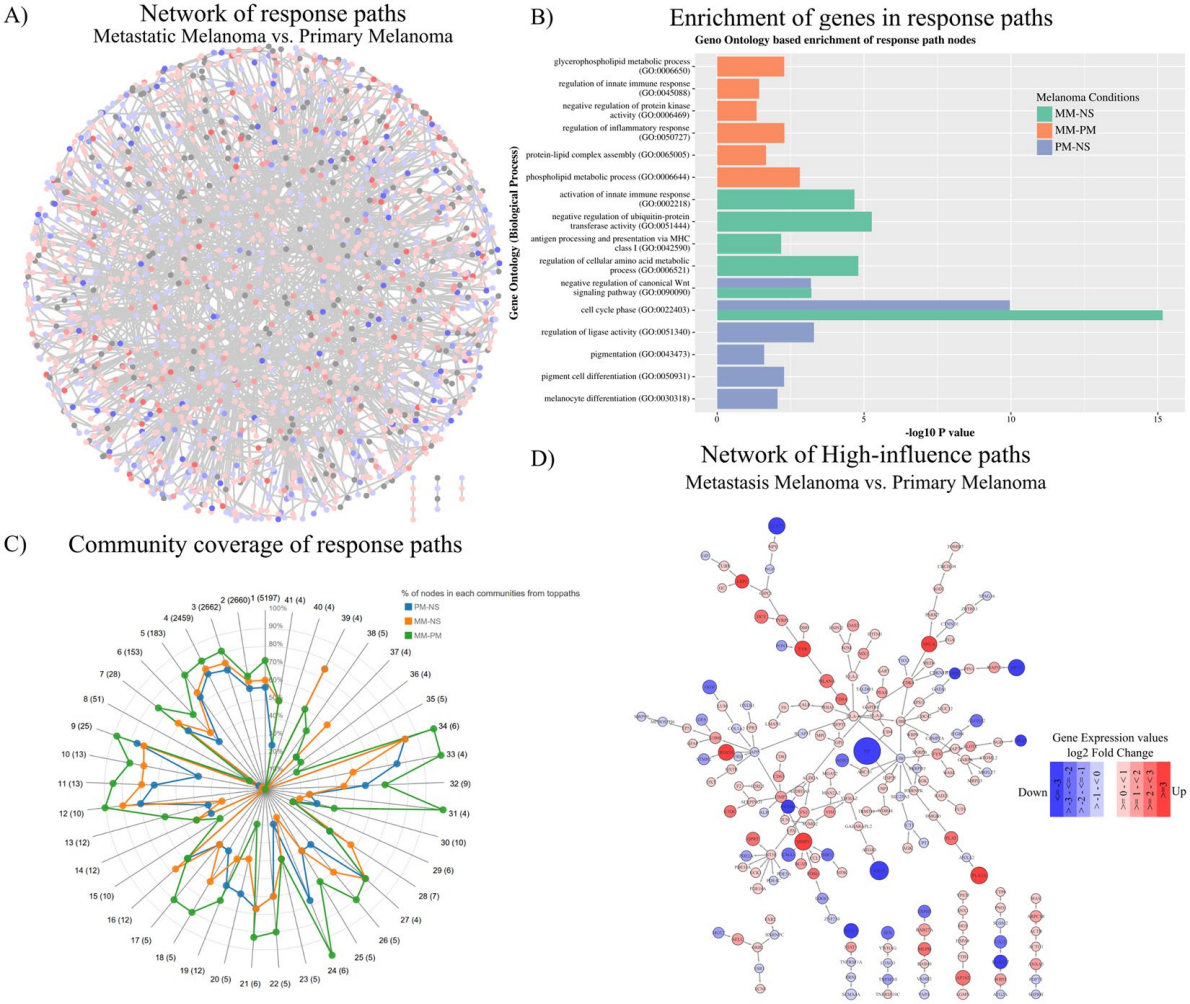
(**A**) A network view of response paths identified using the Jaro-Winkler metric for the MM vs. PM comparison. (**B**) Functional enrichment of differentially regulated genes in top-response paths of MM vs. PM, MM vs. NS and PM vs. NS. (**C**) Percentage coverage of genes in 41 communities by genes of top-response paths from 3 comparisons. (**D**) A subnetwork of response paths of MM vs. PM prioritized based on influence score.

The paths abstracted as strings were compared (MM vs. PM, PM vs. NS, MM vs. NS), using a string similarity metric, that provided a measure of dissimilarity among the three conditions (see Methods). Highest scoring paths in each comparison reflect the set of highest perturbations in the network. We use the term ‘highest perturbations’ to describe the top ranked difference paths in the given pair of conditions (Supplementary Figure S1). A total of about 188 million paths were computed for each condition, of which about 19.5% paths of MM were unique to paths of PM. Similarly, 15% paths of PM and 21% paths of MM were unique when compared to NS. Higher the dissimilarity score, higher are the differential activities and hence the paths were sorted on this basis. We selected only the top ranked 0.001% of paths consisting of ~50% DEGs for further analysis amounting to 36952, 28338 and 39558 paths in the three comparisons MM vs. PM, PM vs. NS and MM vs. NS, respectively. A stringent threshold of 0.001% was used to obtain a shortlist containing sufficient number of promising candidates for taking them further in the pipeline, while minimising chances of false-positives. In each case, although only a small fraction of paths was selected, we observed that these paths form a well-connected subnet. The fact that they are connected subnets strongly suggests that the perturbations are not random in nature and appear to be orchestrated as a system’s response to melanoma. Thus these paths of highest perturbations in a MM vs PM comparison defines the systems’ response to progression of disease from a primary melanoma to a metastatic form and referred to as response paths. Likewise, the paths for PM vs NS and MM vs NS represent the systems’ response for primary melanoma with respect to normal skin or metastatic melanoma versus normal skin respectively. The response paths can also be viewed as highest differences in ‘flows’ in the network, where a ‘flow’ implies a transfer of effect through the path containing differentially regulated genes. The paths, in addition to differentially expressed genes, contain bridging genes that may be constitutively expressed at high levels, and also hub nodes that serve as the main link to multiple flows. The response networks were constructed using the response paths and consisted of 9975 (MM vs. PM), 8457 (PM vs. NS) and 8899 (MM vs. NS) nodes. The potential of such response networks to identify top perturbations and a common core in disease-specific networks has been explored previously^28,29^. A response network of PM vs. NS is shown in Fig. 2A. This exercise resulted in elimination of 49% of DEGs, resulting in a list of 472 DEGs between MM and PM for further processing. Similarly, in the other two comparisons 56% (PM vs. NS) and 53% (MM vs. NS) of DEGs were eliminated. A point to note is that the protein-protein interactome is likely to be incomplete, since many interactions may not even be characterized in any system and it is therefore possible to miss some promising DEGs at this step. This however is not a major limitation in our study, as our goal is to identify biomarkers with high discriminative power rather than evaluate all possible markers.

#### Functional enrichment analysis of the response networks

To gain insights about the functional categories of the genes in the response networks (foreground set), a gene enrichment analysis was carried out against all human genes (background set). The predominant biological processes of each of the response networks are illustrated in Fig. 2B (Supplementary Table S2). For the MM vs. PM set, several processes linked to metastasis such as the phospholipid metabolic process (P = 1.5 × 10^−3^), protein-lipid complex assembly (P = 2.1 × 10^−2^), regulation of inflammatory response (P = 5.1 × 10^−3^), negative regulation of protein kinase activity (P = 4.5 × 10^−2^) and regulation of innate immune response (P = 3.7 × 10^−2^) were enriched. On the other hand, the processes of melanocyte differentiation (P = 8.8 × 10^−3^), pigment cell differentiation (P = 5.2 × 10^−3^), pigmentation (P = 2.5 × 10^−2^), regulation of ligase activity (P = 5.08 × 10^−4^), and cell cycle phase (P = 1.9 × 10^−10^) are the most enriched processes in the PM vs. NS set. Overall, the enrichment analysis indicated that the cell cycle, immune process and processes related to metastasis are prominent in metastatic melanoma, whereas the pigmentation processes was predominantly only in the case of PM but not in MM, as reported earlier by Raskin *et al*.^10^ Another GO process, the negative regulation of the canonical Wnt signaling pathway (P = 6.4 × 10^−4^) was also present in both PM vs. NS and MM vs. NS enrichment. In addition, the metastatic condition has a high enrichment of genes related to lipid synthesis (P = 2.1 × 10^−2^), consistent with the report of Baenke *et al*. for various cancers^31^. *SPP1* (osteopontin), a gene involved in melanoma invasion and tumour progression^32^ is increased by 8-fold in MM compared to PM and is present in MM vs. PM response paths. In an earlier work, we have reported that *SPP1* differential expression increased hazard of death^33^. *MITF*, *RAC1*, *PTEN* and Jak-Stat pathway proteins (*STAT1* and *STAT3*) are some of other proteins involved in invasive and metastatic behaviour of malignant melanoma that are part of top-response networks^34^.

### Screening for High influence genes in the response networks

The genes in the response network were further prioritized based on the extent of influence they wielded in the whole network. The network communities are densely connected subnets of the whole network and are involved in performing similar or interrelated biological functions^35,36^. Functional perturbations to the nodes percolate effectively due to high connectedness within a community. Based on the network topology, we identified 41 communities in the master network. We tested if the nodes in each response network showed good coverage of the communities and observed that most communities (87%) were indeed well covered (Fig. 2C), and hence it was meaningful to use community-spanning to identify the most-influential nodes in the response networks. We then score the paths in each top-response paths based on the number of communities they span using two scoring schemes: (a) the paths consisting of nodes that belong to maximum communities - *max-span* paths (the top 1% of paths spanning the largest number of communities) and (b) the paths with nodes belonging to a single community - *min-span* paths (the top 1% of paths within a community). Max-span paths are enriched with differential genes across multi-function communities while min-span paths are enriched with genes that are most important within a community.

The next filter in the biomarker identification pipeline retains only the highly influential paths and eliminates the rest. For this, we computed an *influence score* (equation 1) for each node to capture the extent of its topological importance in the network and the gene expression variation in the given condition, and thus obtained a measure of a *consolidated influence score* (equation 2) for each path. Top 1% of *max-span* and *min-span* paths ranked based on *consolidated influence score* in each condition were selected and high-influence networks were built. 72 paths (271 edges, 259 nodes) make the high-influence network of MM vs. PM (Fig. 2D). Similarly, 56 paths (192 edges, 194 nodes) for PM vs. NS and 78 paths (278 edges, 263 nodes) for MM vs. NS form the high-influence networks (Supplementary Figure S3 and Table S3). 25 DEGs from the high-influence network were identified as possible candidates to discriminate MM from PM. Similarly, 54 and 104 DEGs were identified for PM vs. NS and MM vs. NS, respectively. These form the first version of the signature panels in each case (Supplementary Table S4).

### Optimization of the panel length and performance evaluation

The signature genes were derived based on median expression values which are oblivious to the heterogeneity of the disease. Given that high extents of heterogeneity are typically observed among patients with the same clinical presentations, it becomes necessary to use a panel of genes. The next question therefore is to identify how many and which genes should constitute the panel to achieve high discrimination in multiple datasets. Towards this, the relative importance of each gene, when treated as a feature was computed in the present dataset (GSE15605). The feature ranking and the receiver operating curves (ROC) from a random forest classifier are shown in Supplementary Figure S4. *KRT16*, a regulator of innate immunity in the skin, significantly downregulated in MM was found to be the highest discriminator between MM and PM. *ALDH1A1*, *IRX4*, *REST*, *WNT3A* and *SPRR3* were the other top ranked genes (full list of all condition comparisons in Supplementary Table S5). Further, we retained only those genes that showed consistent differential expression in another independent transcriptome dataset of 14 PM, 40 MM and 4 NS samples (GSE7553).

**Figure 3:**
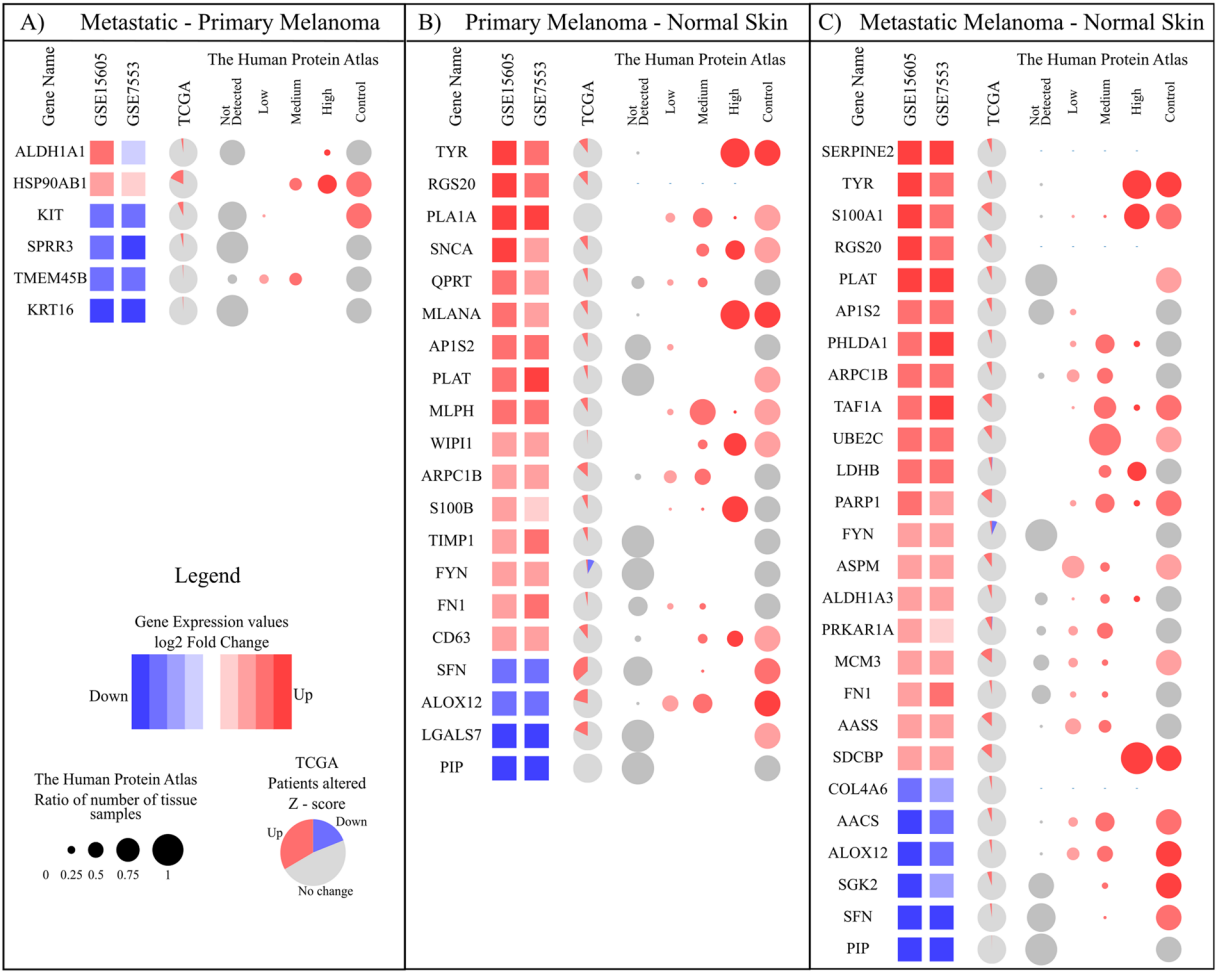
Final signature genes for 3 condition comparisons. (**A**) 6 genes of MM vs. PM (**B**) 20 genes of PM vs. NS (**C**) 25 of MM vs. NS. The first two columns after gene name show the differential expression level in cohort GSE15605 and GSE7553, respectively. Third column show Venn diagrams indicating percentage of patients, the gene is differentially regulated in TCGA based on z-score. In the human protein atlas section, first four columns show the antibody stain levels observed in melanoma tissue. The size of each circle is based on a ratio of the number of patients showing particular expression to the total patients and the colouring is based on the intensity of expression. The last column is stain intensity in control tissue.

We thus identified a final panel of 6 genes (*ALDH1A1*, *HSP90AB1*, *KIT*, *SPRR3*, *TMEM45B* and *KRT16*) which achieved a classification of 87% for MM vs. PM, a panel of 20 genes achieved a classification of 95% for PM vs. NS and 96% by a panel of 25 genes for MM vs. NS. Figure 3 provides a comprehensive illustration of how each gene fared in the two datasets based on gene expression values. In addition, we compared the gene-expression fold change patterns for each gene with the available protein expression levels in melanoma tissue (no stage-specific data was available) and normal skin (Fig. 3), which showed reasonable agreement for many genes. The protein abundances were obtained from the human protein atlas, which were based on antibody staining of the melanoma tissue. The panel is intended as a RNA-signature and hence differential proteomic data is not directly relevant. However, understanding the trend in protein abundances can provide insights towards a mechanistic understanding of the role of the individual gene products, in disease progression and lend support for the selection of biomarkers. Of the 6 markers, *ALDH1A1* (P = 4.3 × 10^−7^) and *HSP90AB1* (P = 4 × 10^−3^) are upregulated in 75% and 83% of patients respectively, while the other 4 genes *KIT* (P = 2 × 10^−3^), *SPRR3* (P = 3.2 × 10^−6^), *TMEM45B* (P = 4 × 10^−3^) and *KRT16* (P = 3.2 × 10^−6^) are downregulated in around 80% patients. We compared the discriminatory power of our panel with that of a similar-sized panel identified without the use of networks, based on only machine learning approach (Supplementary information, Table S1 and Figure S2), which showed that the network based methods have a distinct advantage in identifying the best panel and also contains biologically meaningful genes.

**Figure 4:**
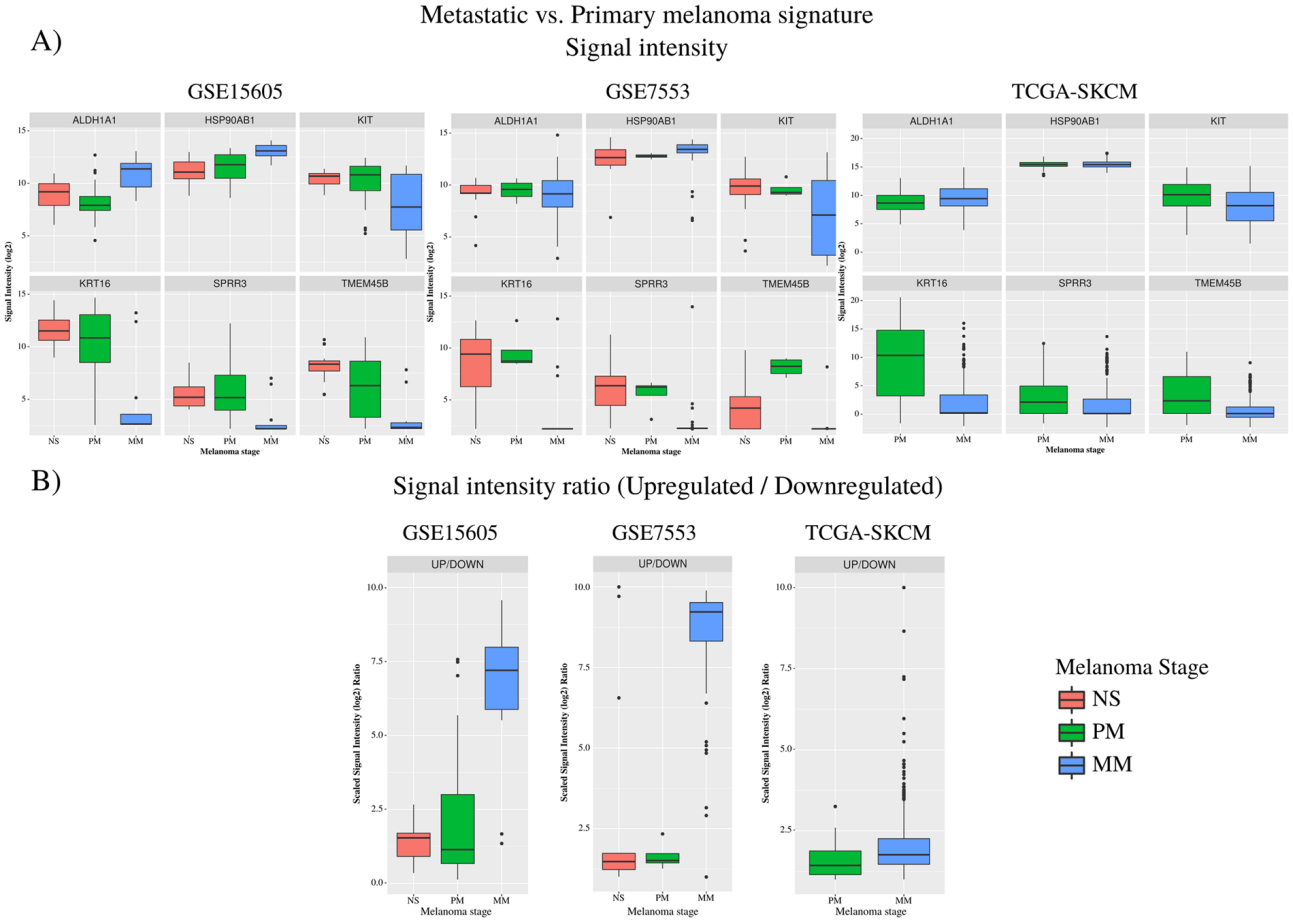
(**A**) log2 intensity values of the 6 genes for each condition in GSE15605, GSE7553 and TCGA. (**B**) The combined score which is computed as a ratio of product of the signal intensity (log2) of upregulated genes (*HSP90AB1* and *ALDH1A1*) to the product of the signal intensity (log2) downregulated genes (*KIT*, *KRT16*, *SPRR3* and *TMEM45B*).

Figure 4A shows the log2 intensity values of these 6 genes for each condition in GSE15605, GSE7553 and TCGA. Figure 4B is ratio of upregulated genes expression product (*HSP90AB1* and *ALDH1A1*) to the downregulated genes expression product (*KIT*, *KRT16*, *SPRR3* and *TMEM45B*) among the 6 gene signature for the 3 cohorts and shows a good separation between the MM and PM. The combined effect size of the panel is seen to be very high in the first two datasets. A clear interpretation is difficult from the TCGA dataset, although the combined score is still higher in MM as compared to PM, because the dataset that is publicly accessible is a pool of samples of known primaries and metastatic samples of unknown primaries, and those collected from different tissues including from lymph nodes, but not individually annotated beyond the broad classification of ‘primary’ and ‘metastatic’ conditions’. The first two datasets on the other hand are more clearly annotated and the samples are all from the skin samples with known primaries.

### Biological significance of the identified panel

To understand the significance of the identified genes, we first analysed how our signature fares with respect to expression of genes known to be differentially expressed in melanoma: tyrosinase (*TYR*), S100 family proteins, *PMEL*, *MLANA*, *MITF*, *FN1*, *LDH*, *S100B*, *MIA* and *CSG4* ^37,38^. From our analysis, we identified that 10 such genes are found either in our PM vs. NS or MM vs. NS signatures. We provide a full list of genes in Table 1, along with their functional categories and their role characterised in melanoma or other cancers. 16 genes in our signatures such as *QPRT*, *ALOX12* and *PIP* are seen to be either known or potential markers of other cancers, but not previously identified in melanoma.

**Table 1:** Biological significance of PM vs. NS and MM vs. NS signature.

Gene symbol		Description	Association with cancer			GO biological process	Remarks
			A	B	C		
**Genes in PM vs. NS**							
TYR*	↑	Tyrosinase	✓			pigmentation	Well established biomarker of melanoma^9^
RGS20*	↑	Regulator Of G-Protein Signaling 20			✓	cell differentiation	Involved in cancer cell aggregation, migration, invasion and adhesion in other cancers^51^
PLA1A	↑	Phospholipase A1 Member A		✓		metabolic process	Identified to be related to short survival of melanoma patients^52^.
SNCA	↑	Synuclein Alpha	✓			metabolic process	Protein of many diseases and also reported as biomarker of malignant melanoma^53^.
QPRT	↑	Quinolinate Phosphoribosyltransferase			✓	metabolic process	A potential marker for follicular thyroid carcinoma^54^.
MLANA	↑	Melan-A	✓				An established melanoma biomarker^37^
AP1S2*	↑	Adaptor Related Protein Complex 1 Sigma 2 Subunit		✓	✓	intracellular protein transport	Upregulated in expression profile of 20 cancer types^55^
PLAT*	↑	Plasminogen Activator, Tissue Type		✓		cell mobility	Plasminogen activation system studied in uveal melanoma^56^
MLPH	↑	Melanophilin		✓		intracellular protein transport	Differentially expressed in melanoma^57^
WIPI1	↑	WD Repeat Domain, Phosphoinositide Interacting 1		✓		metabolic process	Coordinates Melanosome Formation and Melanogenic Gene Transcription^58^
ARPC1B*	↑	Actin Related Protein 2/3 Complex Subunit 1B		✓	✓	cell mobility	Prediction marker for choroidal malignant melanoma and lung cancer^59^
S100B	↑	S100 Calcium Binding Protein B	✓			cell proliferation	An established melanoma biomarker^37,60^
TIMP1	↑	TIMP Metallopeptidase Inhibitor 1		✓		cell proliferation	Timp1 interacts with CD63 to activate PI3-K signaling pathway in melanoma^61,62^
CD63	↑	CD63 Molecule		✓		cell mobility	
FYN*	↑	FYN Proto-Oncogene, Src Family Tyrosine Kinase	✓		✓	cell mobility	Potential biomarker for melanoma and other cancers^63,64^.
FN1*	↑	Fibronectin 1	✓			cell mobility	Used in a diagnostic assay of metastatic melanoma^65^
SFN*	↓	Stratifin		✓	✓	cell death	Downregulated in melanoma and other cancers^12,66^
ALOX12*	↓	Arachidonate 12-Lipoxygenase, 12S Type		✓	✓	skin development	Biomarker for prostate cancer and also downregulated in melanoma^11,67^
LGALS7	↓	Galectin 7		✓	✓	apoptotic process	Dual role observed in melanoma. Downregulation studied in cervical cancer and gastric cancer^68–70^
PIP*	↓	Prolactin Induced Protein			✓	regulation of immune system process	Biomarker for Breast Cancer^71^
**Genes in MM vs. NS**							
SERPINE2	↑	Serpin Family E Member 2			✓	cell differentiation	Therapeutic target for colorectal cancer^52,72^
S100A1	↑	S100 Calcium Binding Protein A1	✓			cell proliferation	Established melanoma marker^37^
PHLDA1	↑	Pleckstrin Homology Like Domain Family A Member 1			✓	cell differentiation	Expression involved in intestinal tumorigenesis^73^
TAF1A	↑	TATA Box-Binding Protein-Associated Factor 1A				Regulation of transcription	
UBE2C	↑	Ubiquitin Conjugating Enzyme E2 C		✓		cell proliferation	Therapeutic target for melanoma^74^
LDHB	↑	Lactate Dehydrogenase B	✓			metabolic process	Established biomarker of melanoma^75^
PARP1	↑	Poly(ADP-Ribose) Polymerase 1		✓		cell differentiation	Associated with poor survival of melanoma patients^76^
ASPM	↑	Abnormal Spindle Microtubule Assembly			✓	cell differentiation	Has a pro-invasion role in metastasis^77^
ALDH1A3	↑	Aldehyde Dehydrogenase 1 Family Member A3	✓			metabolic process	Identified as marker and target of melanoma therapeutics^78^
PRKAR1A	↑	Protein Kinase A Type 1a Regulatory Subunit			✓	cell differentiation	Overexpression studied in cholangiocarcinoma^79^
MCM3	↑	Minichromosome Maintenance Complex Component 3	✓			metabolic process	Is a possible independent prognostic marker for melanoma^80^
AASS	↑	Aminoadipate-Semialdehyde Synthase			✓	metabolic process	Is an oncogene^81^
SDCBP	↑	Syndecan Binding Protein			✓	cell mobility	Involved in cancer development and progression^82^
COL4A6	↓	Collagen Type IV Alpha6 Chain		✓	✓	cell adhesion	Involved in aggressiveness and metastasis of melanoma and other cancers^83^
AACS	↓	Acetoacetyl-CoA Synthetase			✓	cell differentiation	Low expression studied in tumor tissues^84^
SGK2	↓	SGK2,Serine/ThreonineKinase 2		✓		regulation of cell growth	Dowregulated in melanoma^85^

*Genes also present in MM vs. NS signature. A: Melanoma biomarker B: Studies related to melanoma C: Studies related to other cancers.

Of the 6 gene MM vs. PM panel, *HSP90* (heat shock protein 90) is a well-known marker for melanoma and its expression increases with disease progression^39^, *ALDH1A1* is also a previously suggested marker and also potential target to decrease growth, tumorigenicity and metastasis of melanoma^40^. SPRR family and Keratin family genes were described to be downregulated in metastatic melanoma as compared to primary melanoma^41^, consistent with the trend that we observe, for two members of the family, *KRT16* and *SPRR3*. *KIT*, a downregulated gene in this set has been linked to disease progression and is also being explored as a therapeutic target^42^. Overall, as listed in Table 1, we observe that genes belonging to the following gene ontology categories are upregulated in the PM vs. NS and MM vs. NS panels, (a) pigmentation, (b) cell differentiation, (c) cell proliferation and cell mobility (d) metabolic processes, while some genes related to (e) cell death and (f) skin development are downregulated.

### Assessing disease severity and prognosis in a retrospective study of 703 primary melanoma samples from the Leeds melanoma Cohort

To validate the significance of the MM vs. PM signature, expressions of key genes from the identified network were analysed in the Leeds Melanoma cohort (703 primary tumours) to assess their individual and joint effect on melanoma-specific survival (MSS) as well as their association with melanoma histological characteristics: AJCC stage, Breslow thickness, ulceration and mitotic rate (See Methods).

#### Melanoma specific survival analysis (MSS)

In a univariable Cox model, elevated *HSP90AB1* expression significantly predicted increased hazard of dying from melanoma (HR = 1.9, P = 0.0002) while higher expression of *KRT16*, *KIT* and *TMEM45B* reduced the death hazard (HR = 0.9 and P ≤ 0.05 for all three) (see Table 2). In unadjusted multivariable analysis three genes showed independent effects: *HSP90AB1*, *KRT16* and *SPRR3* (Table 2). In multivariable analysis adjusted for sex, tumour site, age at diagnosis and AJCC stage, only *HSP90AB1* remained significant with unchanged death hazard ratio estimate (HR = 2.0, P = 10^−4^, see Table 2).

**Table 2:** Hazard ratios for MSS for individual genes in the whole dataset^&^.

Gene	Univariable MSS		Multivariable unadjusted MSS		Multivariable adjusted MSS	
	HR	P value	HR	P value	HR	P value
*ALDH1A1*	0.9	0.1	0.9	0.2	0.9	0.3
*HSP90AB1*	1.9	2 × 10^−4^	1.7	0.002	2.0	10^−4^
*KIT*	0.9	0.05	0.9	0.3	1.0	0.2
*SPRR3*	1.03	0.5	1.1	0.03	1.0	0.5
*TMEM45B*	0.9	0.005	0.96	0.4	1.0	0.5
*KRT16*	0.9	0.001	0.93	0.04	0.9	0.07

^&^Death hazard ratio (HR) reflects the change from the baseline of 1.0 each time the gene expression is doubled.

#### Association with melanoma histology

The 4 genes that were associated with MSS in univariable analysis (Table 2) were also significantly correlated with ulceration, mitotic rate and Breslow thickness, and concordantly, AJCC stage (see Table 3). *KIT*, *KRT16* and *THEN45B* are known to be expressed by normal skin appendages or stromal tissue, and hence it is possible that the differential expression of these genes in primary compared with metastatic tissue may represent sampling of those normal tissues in primary disease. Among these 4 genes, as expected, expression of *HSP90AB1* increased with tumour thickness and higher mitotic rate, while the other 3 were negatively correlated.

**Table 3:** Association between each gene and histological features of melanoma.

Gene	AJCC (Pvalue)	Ulceration (Pvalue)	Mitotic rate correlation (P-value)	Breslow thickness correlation(P-value)
*ALDH1A1*	0.4	0.4	−0.04 (0.4)	−0.01(0.7)
*HSP90AB1*	0.03	9 × 10^−4^	0.13 (0.001)	0.2 (1.6 × 10^−5^)
*KIT*	5 × 10^−5^	10^−5^	−0.11 (0.005)	−0.2 (2.3 × 10^−10^)
*SPRR3*	0.7	0.6	−0.08 (0.06)	−0.06 (0.1)
*TMEM45B*	5.5 × 10^−13^	2.2 × 10^−11^	−0.2 (7.9 × 10^−8^)	−0.3 (3.1 × 10^−16^)
*KRT16*	4.8 × 10^−12^	2 × 10^−5^	−0.2 (3.1 × 10^−7^)	−0.3, (1.3 × 10^−20^)

#### Composite gene expression score and MSS

**Figure 5:**
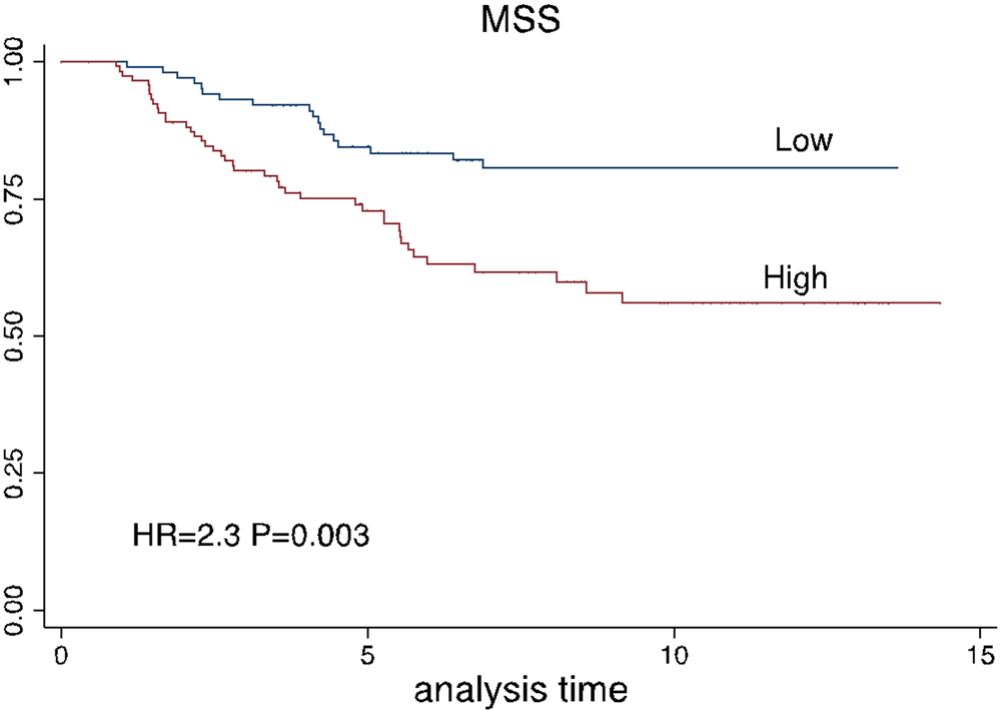
Survival curves according to the combined 6-gene score (unadjusted) in test data (1/3 of total sample). The score was dichotomised by the median.

A composite score was created using expressions of *ALDH1A1*, *HSP90AB1*, *KIT*, *SPRR3*, *TMEM45B*, *KRT16* in the training dataset (random 2/3 of the total dataset, see Methods). When dichotomized on median and applied to the test data (remaining 1/3 of the data), higher values of score predicted worse prognosis with HR = 2.3, P = 0.003 (Fig. 5), remaining prognostic upon adjustment of sex, tumour site, age at diagnosis and AJCC stage with HR = 2.0, P = 0.01.

#### Composite gene expression score and tumour histology

**Figure 6:**
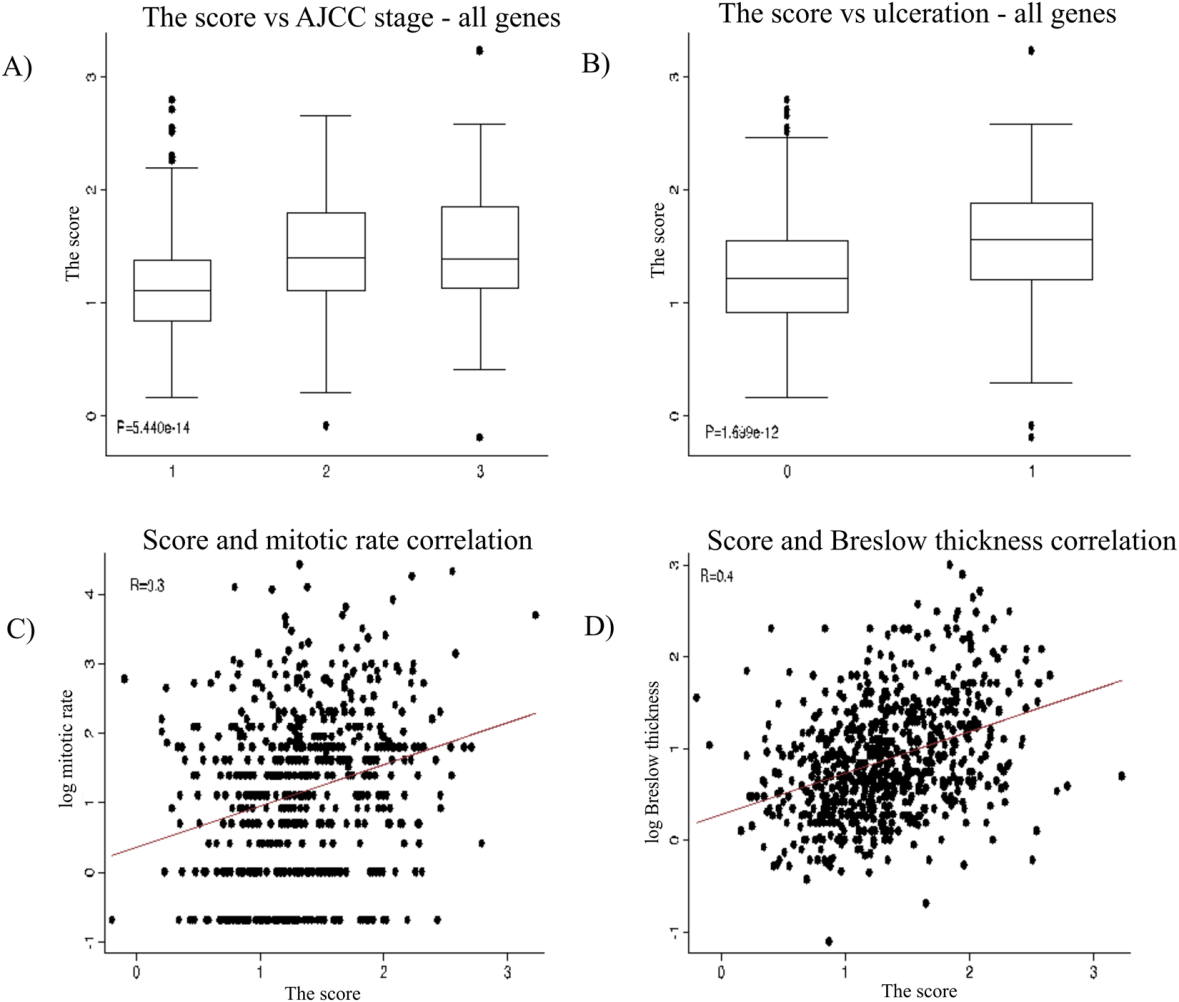
The 6-gene score distribution by AJCC stage (**A**), ulceration status (**B**), mitotic rate (**C**) and Breslow thickness (**D**). Note the log scale for mitotic rate and Breslow thickness.

The score was significantly lower in samples derived from patients with stage 1 (P = 5.4 × 10^−14^) but there was no difference between stages 2 and 3 (Fig. 6A). Tumours with the higher scores were more likely to be ulcerated (P = 1.7 × 10^−12^, Fig. 6B). Mitotic rate and Breslow thickness positively correlated with the score (see Fig. 6C and D).

The scores obtained by removing up to 3 genes (firstly *ALDH1A1*, then *ALDH1A1* and *KIT* and lastly these two and *TMEM45B)* did not significantly change the MSS results (Supplementary Figure S5). All the scores were comparable to the initial score and remained significant after adjustment. The score utilizing the three genes (*HSP90AB1*, *SPRR3* and *KRT16*) was shown to be as strong as the initial 6-gene score in terms of predicting MSS.

Because *KRT16* is highly expressed in the epidermis and its expression may not be entirely from tumours in our data, we further eliminated it and recalculated a score combining only the 2 remaining genes (*HSP90AB1*, *SPRR3)*. This new score remained associated with MSS in the test data (Supplementary Figure S5D).

Thus, from the survival analysis, *HSP90AB1* expression significantly predicted reduced survival (HR = 1.9, P = 2 × 10^−4^ in multivariable analysis and the result remained significant after the adjustment of confounders (HR = 2, P = 10^−4^). Expression of this gene was associated with higher likelihood of ulceration (P = 9 × 10^−4^), higher AJCC stage (P = 0.03) and it was positively correlated with mitotic rate (R = 0.13) and Breslow thickness (R = 0.2), which is concordant with MSS results.

Higher fold change (downregulated) of *KIT*, *KRT16* and *TMEM45B* predicted better prognosis in univariable analysis, however the result did not remain significant in multivariable analysis adjusting confounders (Table 2). The expression of those genes negatively correlated with ulceration, thickness, mitotic rate and ultimately AJCC stage, which is consistent with positive prognostic value. The lack of an independent effect of these genes on MSS is explained by this correlation with these other prognostic tumour characteristics. Although *HSP90AB1* correlated with these tumour characteristics as well (see Table 3), its residual effect on MSS remained significant, suggesting a putatively more potent role.

From the second approach, we see that the 6-gene score trained in 2/3 of the data was a strong predictor of MSS in the remaining 1/3, independent of AJCC stage; hence it might be explored as a prognostic biomarker. This independent prognostic effect was observed in spite of the 6-gene score being also associated with classical melanoma prognostic factors (AJCC stage, ulceration, mitotic rate and Breslow thickness). Interestingly, even after eliminating 3 of the 6 genes, the new 3-gene score (*HSP90AB1*, *SPRR3*, *KRT16*) remained strongly predictive of MSS. This suggests that 3 of the 6 genes identified in the protein network analysis may play a key role in melanoma progression. We note that the combined effect of the 3 genes is roughly similar to that of *HSP90AB1* alone, which is consistent with the results from multivariable analysis which singled out this gene as the only significant when melanoma characteristics are adjusted (Table 2). Therefore, the results from our two analysis approaches highlight the importance of *HSP90AB1* in progression of melanoma.

## Conclusions

We developed a pipeline that combines a network approach with machine learning, through which we identified a biomarker signature capable of discriminating metastatic from primary melanoma tumours. The approach is based on constructing condition-specific genome-wide molecular interaction networks that are specific to each condition and subsequently mining the networks to identify nodes most influential in differentiating between disease stages. The signature genes identified by this network approach have been previously suggested as melanoma markers. In addition, our approach also identifies new potential markers. For many of these, there are studies reported in literature, supporting their roles in the pathophysiology. The discriminatory signature between MM and PM comprises a panel of 6 genes, which exhibit a 6 to 7 fold difference in their combined score between MM and PM. Melanoma specific survival (MSS) analysis for these 6 genes showed 3 genes HSP90AB1, SPRR3 and KRT16 to be strongly predictive of survival, of which HSP90AB1 by itself remained significant for predicting risk of disease progression, even after adjusting for confounding variables and hence has an added prognostic value. In addition to the 6-gene panel, our approach also identified two panels of 20 and 25 genes that can discriminate PM from NS and MM from NS, respectively.

## Materials and Methods

### Datasets

Microarray datasets (i) GSE15605, that contain expression profiles of 16 normal skin, 46 primary melanoma and 12 metastatic melanoma samples^10^, and (ii) GSE7553, that contains expression profiles of 14 primary melanomas, 40 metastases, taken from tumor samples from patients and 4 normal skin samples as controls^12^, were obtained from the NCBI Gene Expression Omnibus (GEO) and used for the discovery phase. Additional datasets used for validation are: (iii) Transcriptomic data of 703 primary melanoma patients from the Leeds Melanoma Cohort generated from formalin fixed primaries using the Illumina DASL array (iv) TCGA dataset – 104 primary melanoma and 367 metastatic melanoma, as available through the cBio Cancer Genomics Portal^43^, and (v) The Human Protein Atlas^44^ containing measurements of proteins based on antibody staining from a few melanoma patients.

Leeds-cohort: 2184 participants with primary melanoma were recruited to the Leeds Melanoma Cohort. As previously described, whole genome transcriptomes were generated from formalin fixed samples taken using a tissue microarray needle^14^. Normalization and analysis was carried out in Leeds as described previously.

### Transcriptome analysis

The microarray analysis was carried out using Bioconductor-R (http://www.bioconductor.org/). The raw intensity values for each tissue sample were normalized using the method GCRMA in Bioconductor package affy. eBayes function was used to identify differential gene expression on linear fitted model generated by lmFit from the package LIMMA. The P-values obtained were adjusted for multiple tests using Benjamini-Hochberg false discovery rate method. Genes with adjusted P-value ≤ 0.05 and a fold change of ±2 were considered as differentially expressed genes (DEGs).

### Protein-protein interaction network

A master human protein-protein interaction network curated earlier in the laboratory^28^ was used. The master network, where proteins were considered as nodes and interactions as edges, consists of 17015 nodes and 200361 edges, of which nearly 80% were directed edges and the rest taken as bi-directional edges (Supplementary information). The available transcriptome data for melanoma GSE15605, mapped onto 21209 genes and finally the corresponding protein-protein interaction network of melanoma genes consisted of 13733 proteins and 179403 interactions.

### Construction of condition-specific interaction networks

The network was rendered condition-specific by integrating it with the gene-expression profile of that condition. The nodes in the network were assigned weights based on the fitted normalized signal intensity values of all genes of NS, PM and MM condition, thus obtaining three networks. The edge between two nodes was weighted as the inverse of the product of the node weights making it compliant with Dijkstra’s algorithm.

### Identification of response paths for each comparison

Response paths are the paths that are highly perturbed between two conditions. Between all-vs-all nodes, high-activity paths were computed using Dijkstra’s algorithm implemented in python-igraph^45^, on condition-specific interaction network of both conditions. The high-activity paths between any two nodes (source and target) in a network were modelled as the linear combination of genes through which information flows with minimal resistance. A path between two nodes is termed ‘perturbed’ if the nodes used to transmit information between the source and target was altered between the two conditions being compared. The paths were considered as strings and the path deregulation was captured using the Jaro-Winkler (JW) distance, a string matching metric^46^ (Supplementary File). The Jaro-Winkler score was normalized between 0 and 1 with 0 indicating an exact match. The top 0.001% perturbed (dissimilar) paths between two conditions were selected using this metric and considered further as response paths.

Functional enrichment analysis was carried out using web-based tool PANTHER – Protein Analysis Through Evolutionary Relationships. The P-values are FDR corrected using Bonferroni correction and considered significant if adjusted P-value < 0.05^47^.

### Identification of high-influence paths

Identification of paths that have the highest influence in the network involved two steps, the first to detect communities or clusters in the network and the second to compute the influence of paths based on the span of these paths across communities and influence wielded by each node in these paths on the entire network.

#### Community detection for the network

Communities were computed using an unweighted, master PPI network to identify the span of paths and reduce the total number of paths based on their efficiency to percolate effect of differential expression. For detecting communities, the Fast-greedy algorithm^48^ implemented in igraph^45^ was used as it is proven to reflect biological network properties efficiently over other community detection methods^49^. A minimal node size ≥4 was imposed to consider a community, which yielded 41 communities, which were taken through further steps in the pipeline.

#### Max-span and Min-span high influence paths

The response paths overlapping on maximum communities were classified as *max-span* paths, which reflect high inter-community influence and the response paths assigned to a single community were classified as *min-span* paths and reflect high intra-community influence. Further, an *influence score* was computed for each node in the *max-span* and *min-span* paths. The score is a combination of the differential expression of the node and its topological position in the network. The *influence score* of node *v* is given as:

1 $$Influence\,scor{e}_{v}=Fold\,chang{e}_{v}\times \frac{D{C}_{v}+{E}_{v}+B{C}_{v}}{3}$$

Where,

DC (Degree conserved), E (eccentricity) and BC (betweeness centrality) values were computed using functions in python-igraph (See supplementary file).

#### Consolidated Influence score

After obtaining an influence score of each node in the *max-span* and *min-span* paths, a *consolidated influence score* was computed for each path.

2 $$Consolidated\,Influence\,scor{e}_{p}=\frac{{\sum }_{i=1}^{n}Influence\,scor{e}_{i}}{n}$$

Where, *n* = number of nodes in path *P*.

It is the sum of influence scores of all nodes in the path, normalized by the path length. The *max-span* paths and *min-span* paths with high score were finally shortlisted to generate a high-influence network for each condition. The DEGs from these high influence networks were further validated for their ability to classify the conditions in a larger dataset.

### Identifying discriminatory genes

From the set of high influence nodes, those that are the most discriminatory between the two conditions in a comparison are then identified. This module involves two steps, the first determines the feature importance and the second prunes the list to finally identify a signature with the best classifying power and the least length.

#### Feature Importance

The ‘extra trees’ classifier was employed to rank important features. The number of estimates was set to 500 while the class weights were automatically assigned so that proportional weights were given to undersampled/oversampled class labels. The criteria for selection was based on entropy (i.e. information gain) and the maximum number of features selected for finding the best split was taken as the sqrt of n_features. The maximum depth was disabled and the nodes were expanded until all leaves are pure.

### Classification accuracy of the final signature

An AdaBoost classifier wrapper was used around the random forest algorithm to compute the classification accuracy of the signature gene set. The number of estimators was set to 500 with a learning rate of 0.08 and the criterion for selection was based on entropy. The boosting function is implemented using the Stagewise Additive Modeling with a Multiclass Exponential loss function (SAMME)^50^. A stratified K-fold was used for cross validation (see supplementary file).

### Validation in the Leeds Melanoma Cohort

Three types of analysis were performed in transcriptomic data in the Leeds Melanoma Cohort using the top ranked network genes:

1. Univariable and multivariable association with melanoma-specific survival (MSS) in a Cox proportional hazards regression, adjusted and unadjusted for patient age at diagnosis, gender, AJCC stage and tumour site.
2. Univariable association of each gene with melanoma histological features using the Kruskall-Wallis test for the categorical variables (AJCC stage, ulceration) and the Spearman correlation coefficient for continuous variables (Breslow thickness and mitotic rate).
3. A composite gene expression score was created by calculating a weighted sum of the expression of each gene. The weight was the univariable log hazard-ratio from MSS analysis in a random selection of 2/3 of the dataset (training set). This score was then applied to the remaining 1/3 (test set) to assess its prediction of MSS and association with tumour histology (AJCC, ulceration, Breslow thickness and mitotic rate). The score’s stability was assessed by removing one by one the genes that had earlier shown the smallest independent effect on MSS in multivariable analyses.

## Electronic supplementary material

Supplementary information

Supplementary Table S2

Supplementary Table S3

Supplementary Table S5
