# Supplementary material for: Identification of a gene signature for discriminating metastatic from primary melanoma using a molecular interaction network approach
Source: Sci Rep. 2017 Dec 11;7:17314. doi: 10.1038/s41598-017-17330-0 (PMC5725601; doi:10.1038/s41598-017-17330-0)

Supplementary Information

Identification of a gene signature for discriminating metastatic from primary melanoma using a molecular interaction network approach

Authors: Rahul Metri[1], Abhilash Mohan[2], Jérémie Nsengimana[3], Joanna Pozniak[3], Carmen Molina-Paris[4], Julia Newton-Bishop[3], David Bishop[3] and Nagasuma Chandra[1, 2*]

Human Protein-protein interaction network

The protein-protein interaction network was built for human genes and the edges rendered directed by manual curation. Experimentally verified PPI with high confidence were collated from various other databases. Below is the list of resources used for constructing the network.

- The Search Tool for The Retrieval of Interacting Genes/Proteins (STRING) (confidence score > 900)
- SignaLink 2.0.4
- The Cancer Cell Map
- BioGRID database
- Multinet

The detailed explanation of PPI construction is described in the article "Sambarey A, Devaprasad A, Baloni P, Mishra M, Mohan A, Tyagi P, Singh A, Akshata J, Sultana R and Buggi S. Meta-analysis of host response networks identifies a common core in tuberculosis. NPJ Systems Biology and Applications. 2017; 3(1):4"

Response Paths

Identification of network paths that are characteristic of disease condition was carried out using well established methods. Figure S1 illustrates the different network terms used in the manuscript.

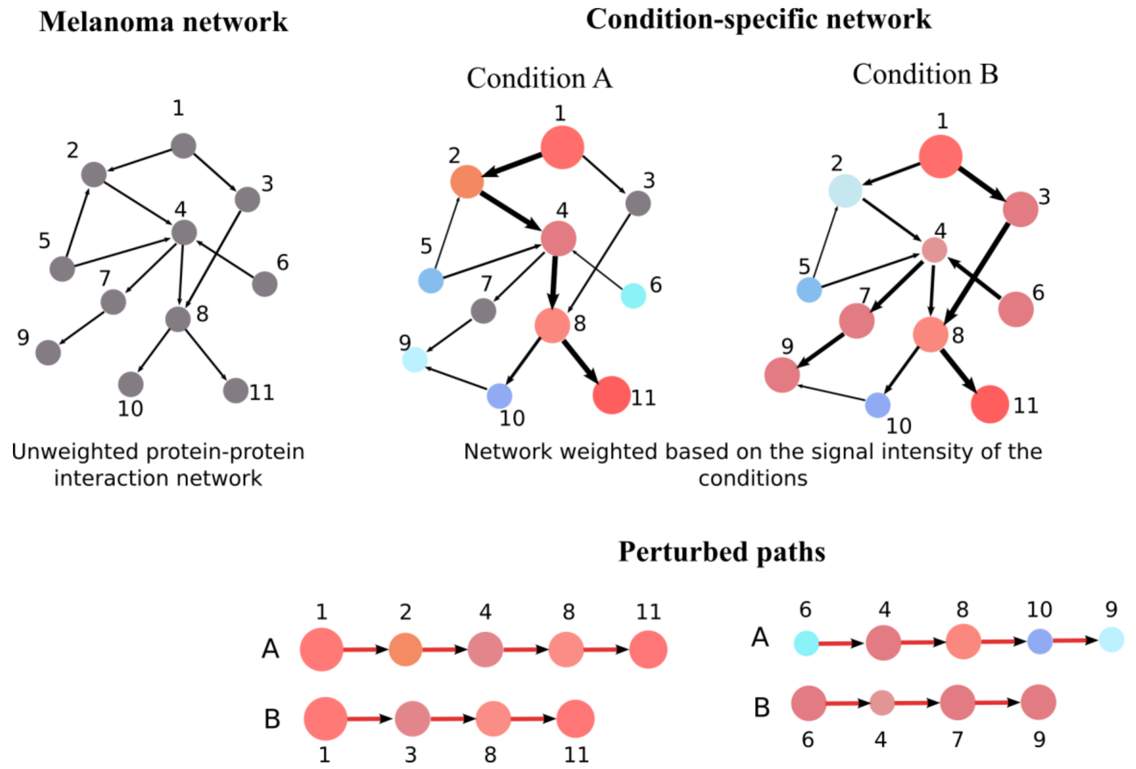

Figure S1: **Identification of response networks**. The unweighted directed melanoma network is weighted using gene expression values of different conditions. Networks of 2 conditions A and B are shown here. Shortest-path computation on A and B identifies top-activity paths from node 1 to 11 and node 6 to 9. Comparisons of these paths between two conditions identifies the paths with maximum difference that are referred to as perturbed paths. The paths with highest perturbations are result of systems' response to progression from one condition to other. The network from such paths form response network. Nodes colors in blue represent low expression levels, red shades represent high expression levels and grey is no expression. Edges width is based on expression weights. High activity paths traverse through highly expressed nodes.

To identify the difference between paths of two conditions, the paths were treated as strings and string similarity was computed. The Jaro-Winkler distance is a string similarity matching metric, originally used to study record-linkage.

$$d_j = \begin{cases} 0 & if\ m = 0 \\ \frac{1}{3}\left(\frac{m}{|s_1|} + \frac{m}{|s_2|} + \frac{m-t}{m}\right) & otherwise \end{cases}$$

Where m is the number of matching characters and t is the half of the number of transpositions while s_1, s_2 are the two strings that are being matched. The criterion for a match between the two strings is given by $\lfloor\frac{max(|s_1|,|s_1|)}{2}\rfloor - 1$. The Jaro distance d_w is given by $d_w = d_j + (lp(1 - d_j))$ where p is a scaling factor (0.1) which gives extra weightage for strings that match from the beginning for a set prefix length l.

Influence score

Degree conserved **(DC)** is a measure where a ratio is computed between the degree of node v in the top-perturbed path network to degree of node v in preliminary melanoma network. Higher ratio signifies that the node and its connections are important for the specific disease or conditions and minimum ratio means the node and neighbors have a low role to play.

$$DC_v = \frac{deg_{\,v\,(top-perturbed\ network)}}{deg_{\,v\,(melanoma\ network)}}$$

Eccentricity (E) is a node centrality index. The eccentricity of node v is calculated by computing shortest paths between node v and all other nodes in the network, then choosing the longest shortest path. Suppose w is the farthest node from v and the length is dist(v,w), eccentricity of the node v is (1/ dist(v,w)). If the eccentricity is high that means all other nodes are in the proximity of the node v and if eccentricity is low then the nodes are far from the vicinity of this node.

$$Eccentricity_v = \frac{1}{max\{dist(v,w)\}}$$

Nodes with high eccentricity will have the longest shortest paths and hence will be linked to proteins that have the highest 'reach' in the network. In biological networks, such nodes are most likely to exert an influence on the highest number of nodes in the network. In contrast, a protein with low eccentricity will have fewer proteins to influence and the overall effect may be minimal.

Betweenness Centrality (BC) is a node centrality index. The betweenness of node v is calculated as the ratio of shortest paths between nodes (m,n) which pass through node v to the total number of shortest paths between the nodes m and n (m,n)

$$Betweenness\ Centrality_v = \sum_{m \neq v} \sum_{n \neq v} \frac{P_{mn}(v)}{P_{mn}}$$

$P_{mn}(v)$: Number of shortest-paths between nodes m and n passing through node v

P_{mn} : Number of shortest-paths between nodes m and n

In biological networks, high betweenness centrality reflects a measure of importance of that node in reaching other distant communicating proteins together.

Machine Learning Methods

Extra Trees algorithm is based on based on randomized decision trees. The algorithm uses the perturb-and-combine technique which is specifically designed for trees. In this method, a diverse set of classifiers are initiated and the creation of these classifiers is achieved by introducing randomness. The prediction accuracy is given as the average prediction of the individual classifiers[1]. This method is similar to the random forests, as in, a random subset of candidate features is used, but the thresholds are picked randomly and the best of these randomly generated thresholds are used as the rule for splitting. The advantage of this method is that it allows in the reduction of variance of the model but on the downside it results in the slight increase in bias[2].

K- Fold

Stratified K-fold[3] is the name given to the method when the stratification process or the rearrangement of data is performed to ensure that the selected fold has approximately similar mean response values this is done to ensure that the stratified data is a representative sample of the whole data. For instance, in a binary classification such as the present problem, each class comprises 50% of the data; hence during the process of rearrangement, it is best to ensure that each class finds almost equal representation is every fold.

Benchmarking of marker identified by networks method compared to machine learning method.

To benchmark our pipeline compared to the standard machine learning approach alone to identify signatures, we carried out following exercise.

Normalized signal intensities of all the genes were used to identify optimal signature set by recursive feature elimination step using the Extra Trees algorithm (see Methods). We considered the top 6 genes (same size as our MM vs. PM signature), which are TMPRSS11B, DPT, LGALS2, ASAP1, ALPI and NEUROG3 as the features.
The classification accuracy estimation results of this 6-gene panel using random forest algorithm (see Methods) is given below

CA	F1	Precision	Recall
0.737	0.1	0.125	0.083

Table S1: The classification report using our ML algorithm against the top 6 genes identified by feature elimination.

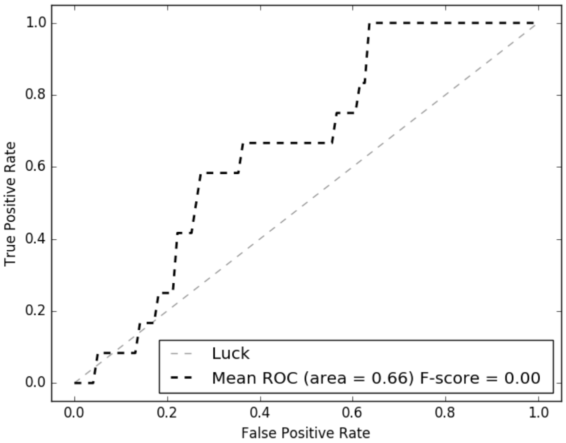

Figure S2. The ROC curve with our ML algorithm using top 6 genes

The classification accuracy of these 6 genes was 73% which is lower than 87% accuracy achieved by signature derived using networks approach. This shows that our pipeline has the highest accuracy in achieving the classification between primary and metastatic melanoma samples.

In addition, an analysis of the functional significance of these genes indicated that only one gene (ASAP1) has an established role in melanoma and the rest are not directly related[4]. This additionally shows the advantage of networks method to obtain the features of biological importance.

References

1. Ho TK. The random subspace method for constructing decision forests. *IEEE transactions on pattern analysis and machine intelligence.* 1998 Aug;20(8):832-44.
2. Geurts P, Ernst D, Wehenkel L. Extremely randomized trees. *Machine learning*. 2006 Apr 1;63(1):3-42.
3. Kohavi R. A study of cross-validation and bootstrap for accuracy estimation and model selection. *InIjcai* 1995 Aug 20 (Vol. 14, No. 2, pp. 1137-1145).
4. Hou T, Yang C, Tong C, Zhang H, Xiao J, Li J. Overexpression of ASAP1 is associated with poor prognosis in epithelial ovarian cancer. International journal of clinical and experimental pathology. 2014;7(1):280.

Supplementary Tables

Table S2: Gene Ontology based enrichment of response network genes (**Supplementary_Table_S2.xlsx)**

Table S3: High Influence Max-Span and Min-Span Paths **(Supplementary_Table_S3.xlsx)**

Table S4: First version of biomarker signatures

**Condition**	**Genes**	**Number of genes**
MM - PM	ALDH1A1;CA12;CCDC113;CDKN1A;CRABP2;FUT8;GTF2F2;HSP90AB1;IRF7;IRX4;IVL;KIT;KRT16;MMP1;MPDZ;NTS;REST;SFRP2;SPRR3;THBD;TMEM45B;WNT4;WNT5A;XRN2;ZBTB6	25 (9 UP; 16 DOWN)
PM - NS	ALOX12;ANXA5;AOX1;AP1S2;ARPC1B;BCAN;BPY2;C1QC;CA12;CCL27;CD63;CD8A;CDK4;CMA1;DCT;DES;EXPH5;FLOT1;FN1;FOSL1;FYN;GSTM5;HBA1;HBB;KRT4;LDOC1;LGALS7;LRP2;MAP1S;MLANA;MLPH;MMP1;MUC1;MUC7;OAS2;OGN;PDE2A;PFAS;PIP;PLA1A;PLAT;PON1;QPRT;RAB27A;RGS20;S100B;SDC1;SFN;SNCA;STAT1;TIMP1;TYR;VIM;WIPI1	54 (32 UP; 22 DOWN)
MM - NS	AACS;AASS;ABCD1;AKR1B1;ALDH1A3;ALDH2;ALOX12;ANXA5;AP1S2;ARPC1B;ASPM;BAX;C1QB;C1QC;C3AR1;CANX;CD63;CD8A;CD9;CDC6;CDK4;CFI;CLU;COL4A1;COL4A6;CRABP2;EBF1;FAM107A;FANCL;FLOT1;FN1;FUT8;FYN;GANAB;GFPT1;GRP;GSTM5;GYPC;HAO2;HBA1;HBB;HLA-B;HSPH1;IL6;ITGB4;IVL;LDHB;LRP2;LRP6;LYST;MCM3;ME2;MMP7;MMP9;MUC1;MUC20;MUC7;MYC;NAT6;NBN;NDRG1;NME7;NR1D1;NR4A2;NTS;OGN;ORC6;PARP1;PDIA3;PHLDA1;PIP;PLA1A;PLAT;PLEKHF2;PNPLA3;POT1;PRC1;PRKAR1A;QPRT;RAD21;RGS20;RXRA;S100A1;S100B;SDC1;SDCBP;SELP;SEMA4A;SERPINE2;SFN;SGK2;SNCA;SNX1;STAT1;STMN2;TAF1A;TIMP1;TRPV1;TST;TYR;UBE2C;VIM;WNT3;ZNF521	104 (67 UP ; 37 DOWN)

Table S5: Rank order of genes based on classification efficiency **(Supplementary_Table_S5.xlsx)**

Supplementary Figures

Figure S3: A) High influence network of MM-NS

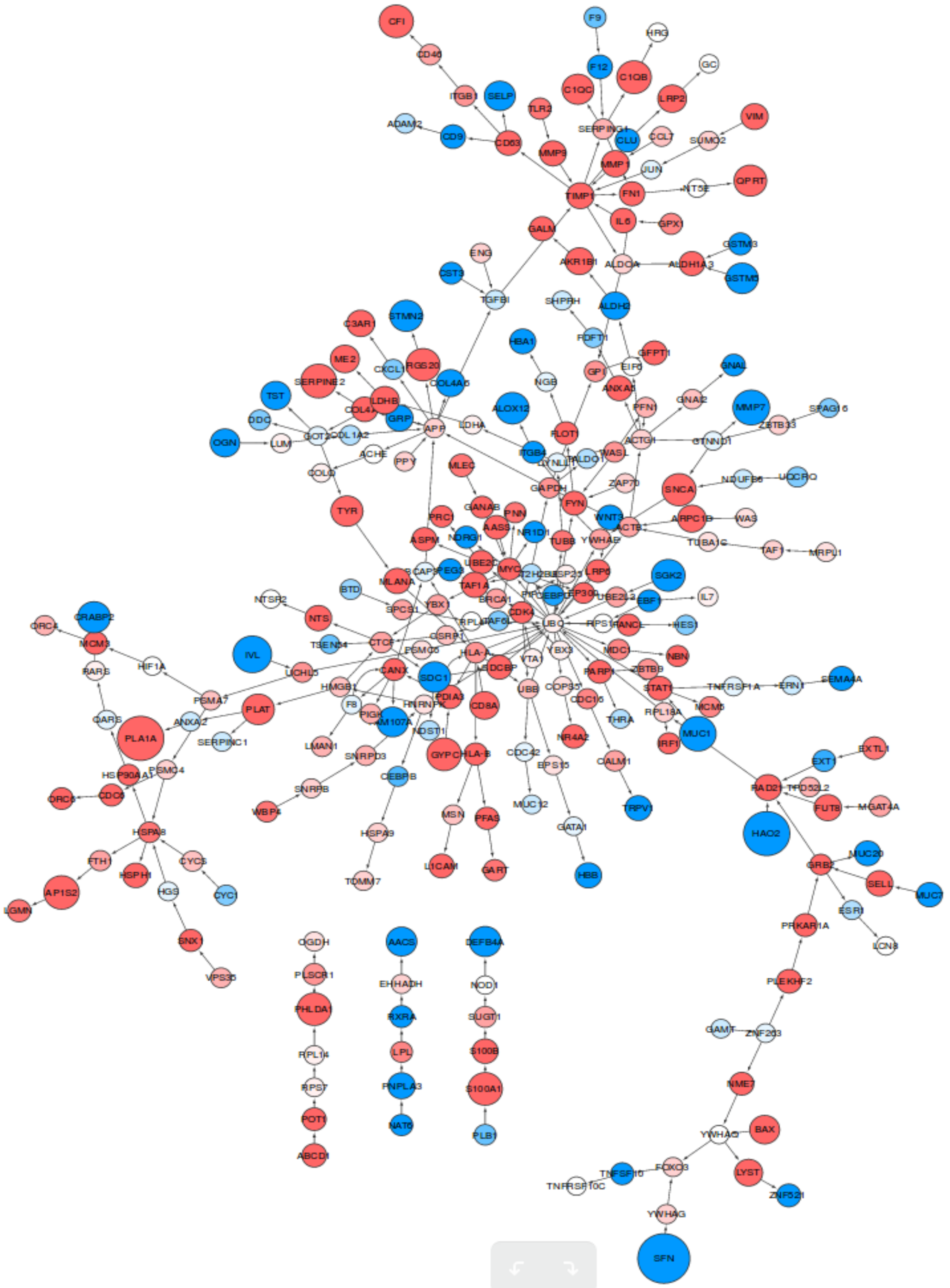

B) High influence network of MM-PM

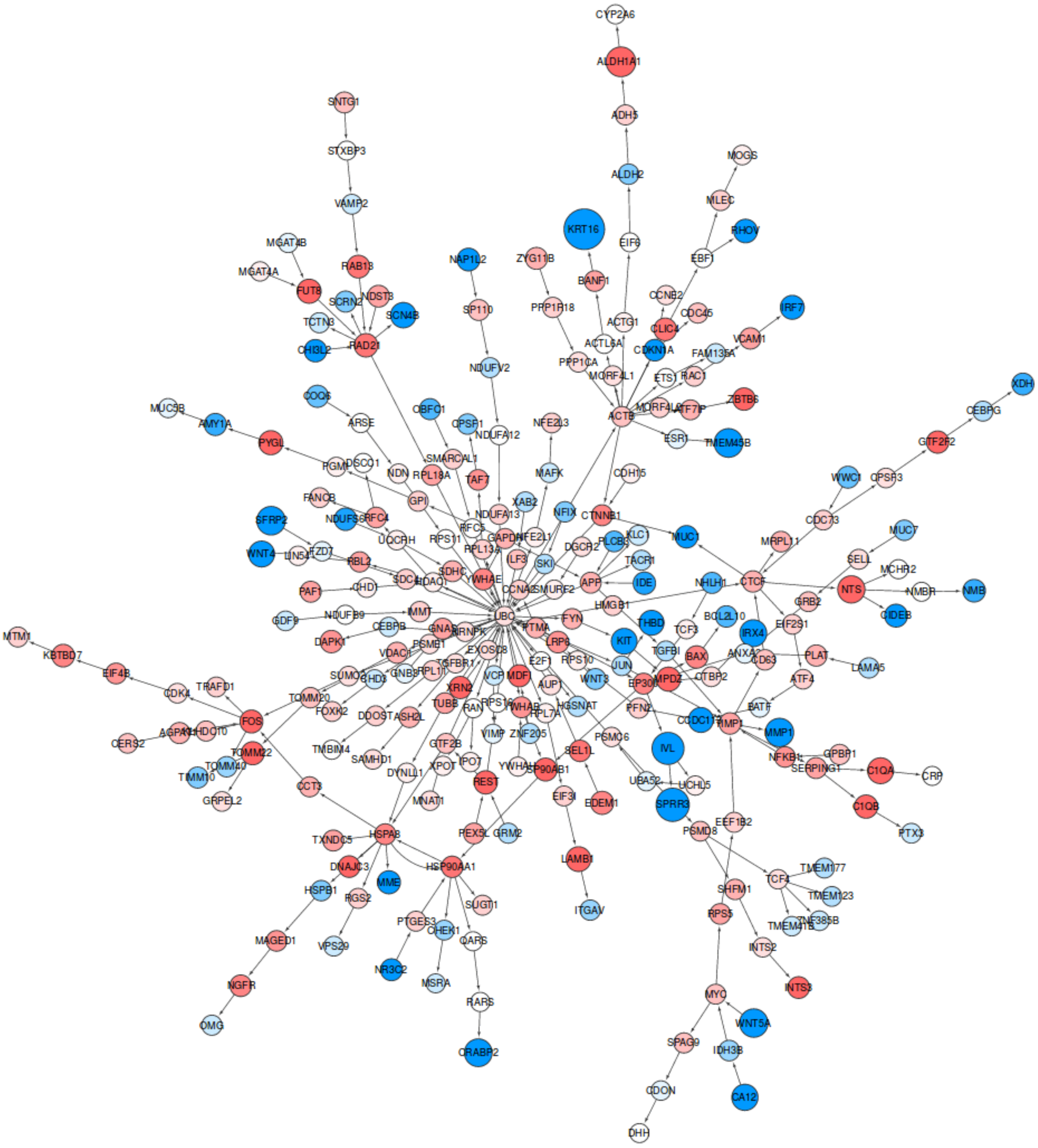

Figure S4: Feature rank order of first version markers in dataset GSE15605

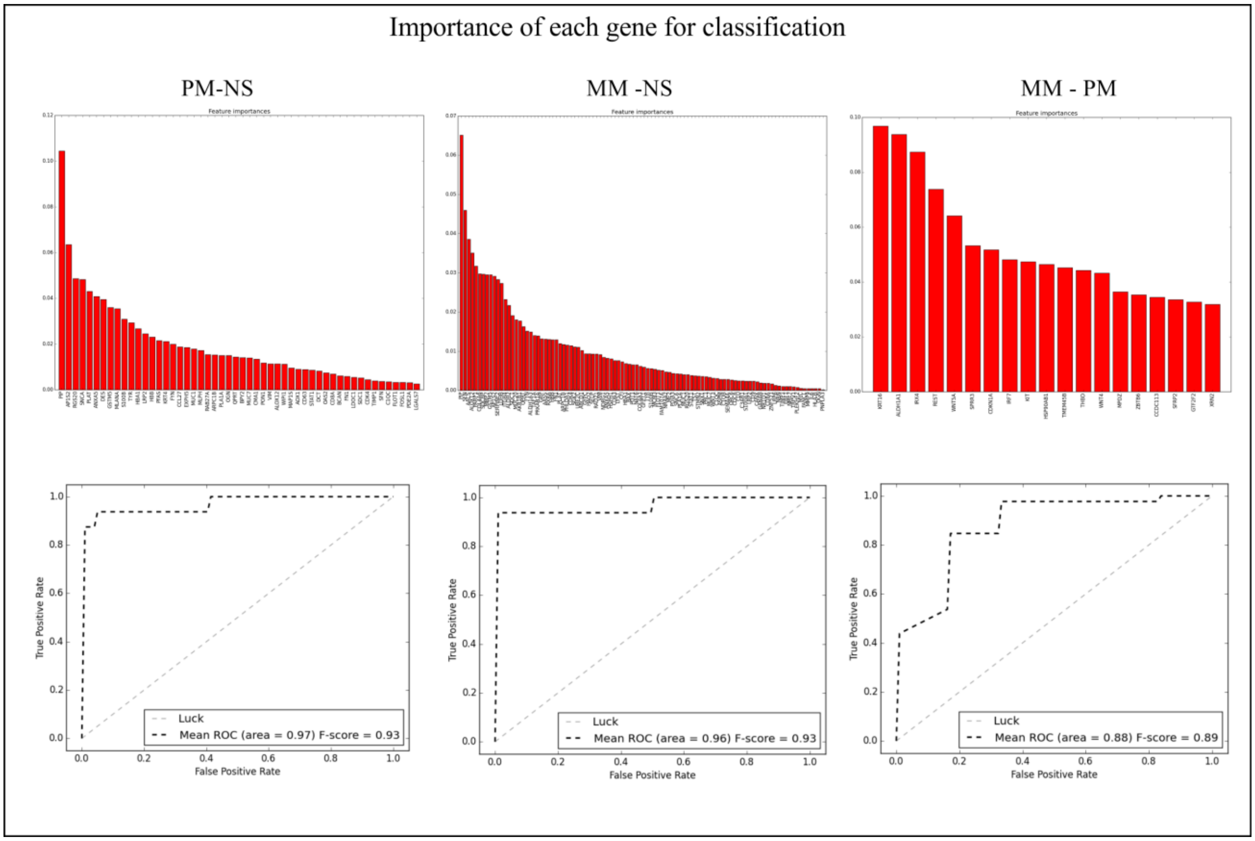

Supplementary Figure S5. Survival curves generated by removing genes progressively which were not significantly associated with MSS in multivariable analyses. The score was dichotomised by median. In **A**. *ALDH1A1* was removed. In **B**. *TMEM45B* and ALDH1A1 were removed. In **C**. TMEM45B, ALDH1A1 and KIT were removed. All the hazard ratios were obtained from unadjusted Cox proportional hazard regression in the test dataset (1/3 of the total sample). The score remained significant after adjustment for sex, tumour site, age at diagnosis and AJCC stage: **A**. HR=2.0, P=0.02; **B**. HR=2.0, P=0.02; **C**. HR=2.3, P=0.03.

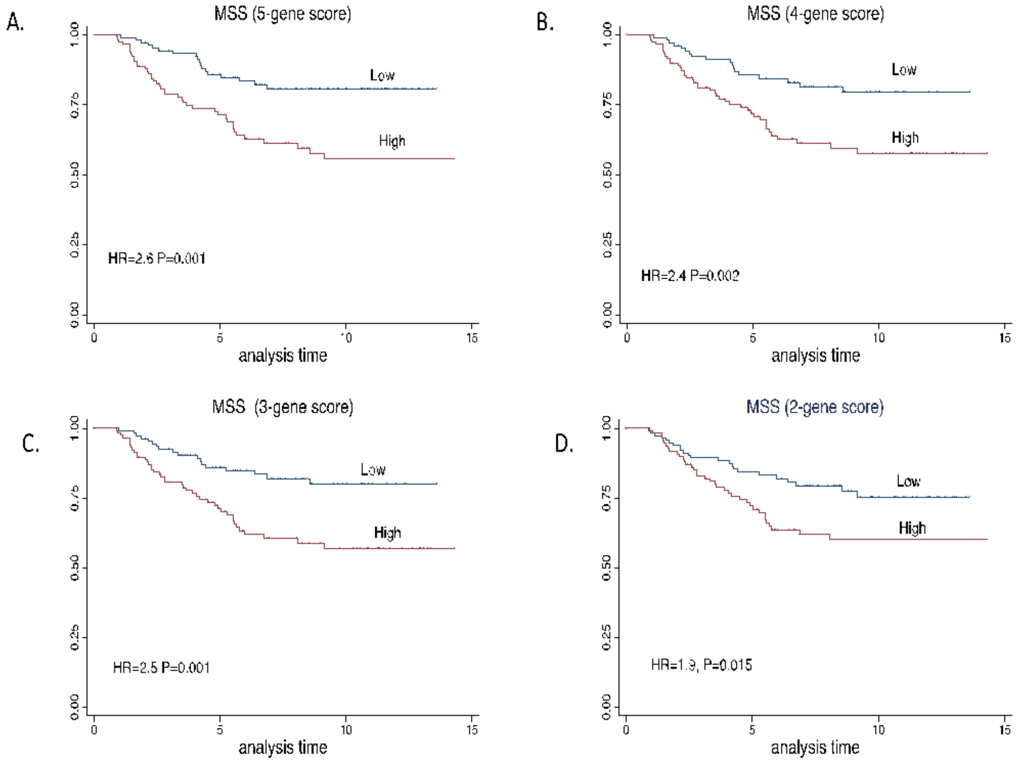

Supplement: Supplementary file 1 — Supplementary information [file 41598_2017_17330_MOESM1_ESM.pdf]
